# Familial Mediterranean fever in Azerbaijani children: a nationwide cohort study of genotype–phenotype association

**DOI:** 10.1093/rheumatology/keag501

**Published:** 2026-09-11

**Authors:** Vafa Guliyeva, Govhar Verdiyeva, Leyla Sahratova, Firengiz Ibrahimova, Aytac Guluzade, Aslıhan Dagdemir, Jale Mammadova, Khariga Jabbarli, Tahmina Karimzada, Rashad Taghiyev, AghaRza Aghayev, Bayram Bayramov, Anar Tagiyev, Khatira Mustafayeva, Konul Haziyeva, Jale Nesibova, Vafa Muslumova, Mirjavid Muslumov, Rashad Abizade, Telman Aytayev, Fatma Gül Demirkan

**Affiliations:** Department of Pediatric Rheumatology, Liv Bona Dea Hospital, Baku, Azerbaijan; Department of Pediatrics, Educational Therapeutic Clinic of Azerbaijan Medical University, Baku, Azerbaijan; Department of Pediatrics, Republican Diagnostic Center, Baku, Azerbaijan; Department of Pediatrics, The Azerbaijan State Doctors Training Institute in honor of Aziz Aliyev, Baku, Azerbaijan; Department of Pediatrics, Aziz Aliyev Central Children’s Hospital, Nakhchivan, Azerbaijan; Department of Medical Genetics, Referans Clinical Laboratory Center, Baku, Azerbaijan; Department of Medical Genetics, Referans Clinical Laboratory Center, Baku, Azerbaijan; Department of Medical Genetics, Referans Clinical Laboratory Center, Baku, Azerbaijan; Department of Medical Genetics, Referans Clinical Laboratory Center, Baku, Azerbaijan; Department of Pediatrics, Republican Pediatric Center, Baku, Azerbaijan; Department of Medical Genetics, National Hematology and Transfusion Center, Baku, Azerbaijan; Genetic Resources Institute, Ministry of Science and Education, Laboratory of Human Genetics, Baku, Azerbaijan; Department of Pediatric Gastroenterology, Leyla Medical Clinic, Baku, Azerbaijan; Department of Medical Genetics, Ellab Diagnostic Center, Baku, Azerbaijan; Department of Molecular Biology and Medical Genetics, Scientific Research Centre, Azerbaijan Medical University, Baku, Azerbaijan; Department of Medical Genetics, Afgen Genetic Diagnosis Center of the Biological Medicine Clinic, Baku, Azerbaijan; Department of Medical Genetics, Shafa Medical Center, Baku, Azerbaijan; Department of Medical Genetics, Shafa Medical Center, Baku, Azerbaijan; Department of Rheumatology, Educational Therapeutic Clinic of Azerbaijan Medical University, Baku, Azerbaijan; Department of Pediatrics, Central Customs Hospital, Baku, Azerbaijan; Department of Pediatric Rheumatology, University of Health Sciences, Kanuni Sultan Süleyman Training and Research Hospital, İstanbul, Türkiye

**Keywords:** Familial Mediterranean fever, genotype–phenotype correlation, phenotype

## Abstract

**Objectives:**

Familial Mediterranean fever (FMF) is highly prevalent in Mediterranean populations, yet data from Azerbaijan remain scarce despite its location within the traditional FMF belt. This study presents the first nationwide characterization of paediatric FMF in Azerbaijan.

**Methods:**

A multicentre retrospective cohort study was conducted across paediatric and genetic referral centres in 11 regions of Azerbaijan. Demographic, clinical and genetic data were analysed. Genotype–phenotype associations were evaluated, with particular emphasis on the distribution of variants across exons and the allelic burden of exon 10 variants.

**Results:**

A total of 349 paediatric patients (60.45% boys) were enrolled. The median age at symptom onset and diagnosis was 4 (0–18) and 7 (0–23) years, respectively. The most prevalent *MEFV* variants were M694V (47.3%), V726A (20.1%) and R202Q (19.8%). Zygosity distribution was 18.3% homozygous, 40% heterozygous and 41.5% compound heterozygous. Genotype–phenotype analyses included 329 patients after excluding isolated R202Q heterozygotes. Fever (79.3%) and abdominal pain (66.6%) were the most frequent manifestations. Compound heterozygous exon 10 genotypes were associated with earlier symptom onset in univariate analyses. In multivariable hierarchical logistic regression analyses, the presence of at least one M694V allele independently increased the likelihood of early-onset disease (odds ratio 2.10; 95% CI: 1.29, 3.43; *P* = 0.003), while the zygosity-based model supported an association between biallelic exon 10 involvement and early disease onset.

**Conclusion:**

This first nationwide paediatric FMF study from Azerbaijan reveals substantial regional genetic heterogeneity, with compound heterozygosity associated with earlier disease onset. These findings advance FMF epidemiology in the Caucasus and support improved genetic counselling and surveillance for high-risk genotypes.

## Graphical abstract

**Graphical Abstract:**
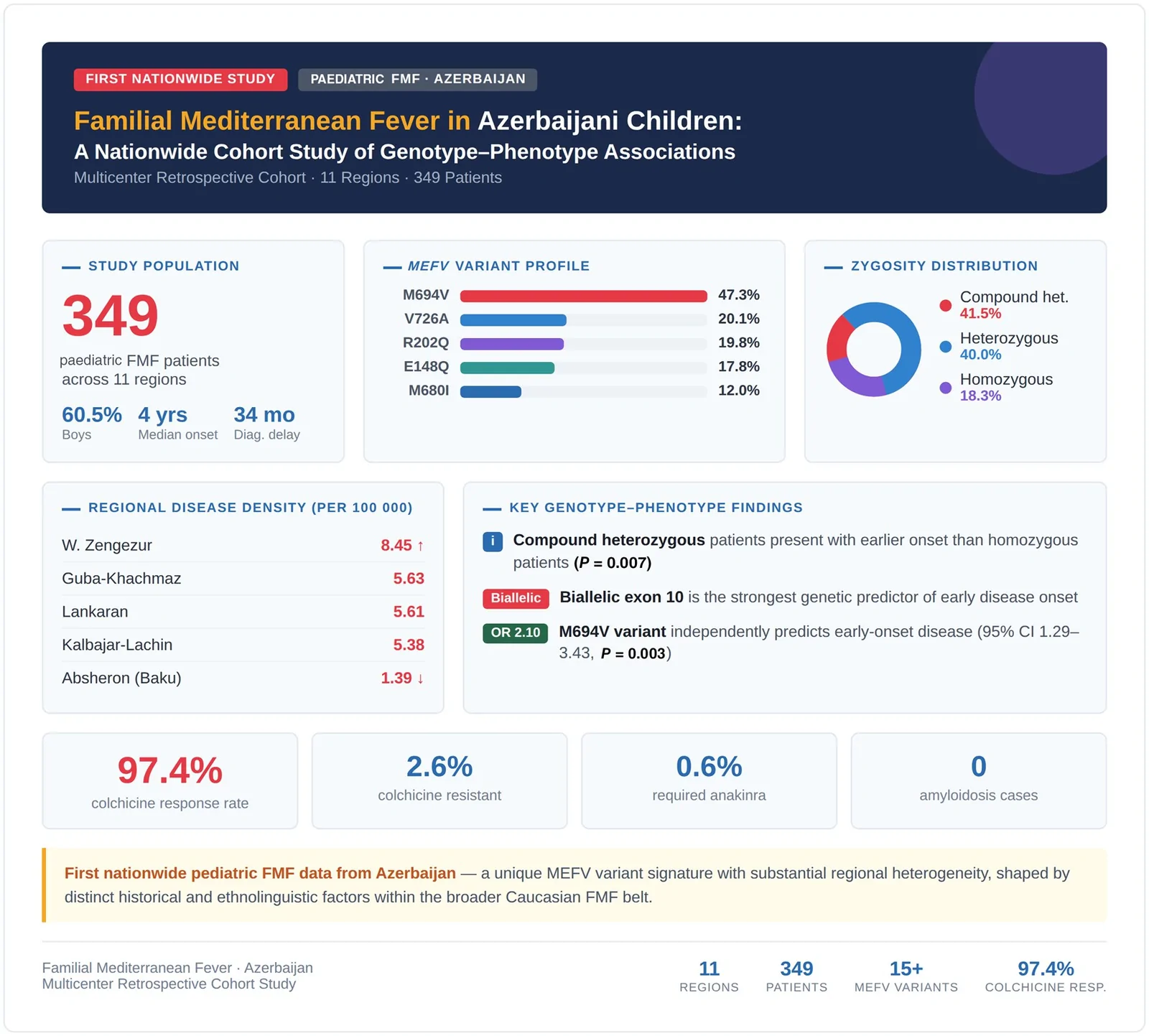

*Rheumatology* key messages

- First nationwide paediatric FMF cohort from Azerbaijan reveals substantial regional genetic heterogeneity.
- Biallelic exon 10 configurations and M694V alleles strongly predict very early-onset familial Mediterranean fever.
- These foundational epidemiological findings improve genetic counselling and personalized healthcare policies across the Caucasus.

## Introduction

Familial Mediterranean fever (FMF) is the most common monogenic autoinflammatory disorder predominantly observed in populations of the Mediterranean basin, such as Turks, Jews, Arabs, Armenians, and Greeks, though sporadic cases have been documented across diverse ethnic backgrounds worldwide [1, 2]. The disease is characterized by recurrent, self-limiting febrile attacks with serosal inflammation [3]. At the molecular level, FMF results from missense mutations in the *MEFV* (Mediterranean fever) gene leading to a pro-inflammatory state, which encodes pyrin, a protein regulating caspase-1 activation and IL-1β production [4, 5]. Dysregulation of this pathway causes uncontrolled inflammation, the pathophysiological hallmark [6]. Over 350 *MEFV* variants have been identified, contributing to significant phenotypic and genotypic heterogeneity [7]. However, the genotype–phenotype relationship in FMF remains incompletely understood. Evidence from large paediatric cohorts shows that exon 10 variants, particularly M694V, are associated with more severe clinical manifestations [8–10]. While diagnosis relies primarily on clinical features, *MEFV* sequence analysis provides essential confirmation [11].

Despite FMF being well-characterized in Mediterranean populations, comprehensive epidemiological and clinical data from Azerbaijan remain notably scarce. This gap is significant given the country’s location within the traditional FMF belt and the genetic admixture of the Azerbaijani population with Near Eastern and Turkic ancestries—groups known for a high prevalence of *MEFV* variants—suggesting an elevated disease risk [9, 12, 13]. Such limited data hinder a clear understanding of FMF burden across the broader Mediterranean and Caucasian regions.

To address these critical knowledge gaps, we performed the first nationwide, multicentre characterization of paediatric FMF in Azerbaijan. Beyond providing a preliminary census, we aimed to define the clinical spectrum and geographic distribution of FMF across eleven major regions, delineate the *MEFV* variant profile using comprehensive exon coverage, and explore genotype–phenotype association. These baseline data will inform healthcare policies in Azerbaijan and contribute to global understanding of genetic and phenotypic diversity of FMF, ultimately enabling improved diagnostic and therapeutic strategies.

## Methods

### Study design and setting

This multicentre study employed a retrospective cohort design and was conducted across major referral paediatric and genetic centres in the Republic of Azerbaijan. Children with a previous diagnosis of FMF who had been followed periodically at the participating centres for at least 1 year prior to study initiation were eligible for inclusion. Retrospective data collection was performed between June 2025 and January 2026 by a multidisciplinary team comprising one paediatric rheumatologist, one paediatric gastroenterologist, three general paediatricians and four clinical geneticists.

### Study population

The study cohort comprised Azerbaijani children aged ≤18 years at diagnosis who fulfilled the clinical diagnostic criteria for FMF according to the Yalçınkaya–Özen/Tel-Hashomer criteria and/or the Eurofever/PRINTO classification criteria [14, 15].

Patients were excluded in cases of incomplete or unavailable molecular analysis of the *MEFV* gene, insufficient clinical documentation or inadequate follow-up data. Individuals who had alternative diagnoses that could confound clinical interpretation were also excluded.

### Clinical and epidemiological data collection

Clinical and follow-up data were systematically extracted from electronic medical records using a standardized data collection form to ensure inter-centre consistency.

Demographic data (age, gender, geographical origin within Azerbaijan) and age of onset of clinical symptoms and age at diagnosis, family history of FMF, clinical manifestations during attacks (fever, abdominal pain, chest pain, arthritis/arthralgia, erysipelas-like erythema), treatment modalities, and adverse effects were recorded for each patient.

Colchicine resistance was defined as the occurrence of one or more attacks per month over a minimum period of 6 months, despite treatment with the maximum tolerated dose [16].

Early-onset FMF was defined as symptom onset at ≤3 years of age, in accordance with the established definition of very early onset disease in the paediatric FMF literature [17, 18].

Administrative districts were categorized into 11 major regions to facilitate a uniform assessment of the epidemiological burden and geographic spread of FMF across the country. To evaluate the epidemiological distribution, disease density was calculated by dividing the observed cases by the regional population data obtained from the State Statistical Committee of the Republic of Azerbaijan (2025) and expressed per 100 000 inhabitants (State Statistical Committee of the Republic of Azerbaijan, Regional population data, last updated September 8, 2025, https://stat.gov.az). This normalization allowed for a comparative assessment of disease prevalence across regions with disparate population sizes.

### *MEFV* molecular analysis and genotype classification

*MEFV* molecular analysis was performed using either Sanger sequencing or next-generation sequencing (NGS), covering all coding exons of the *MEFV* gene. Identified variants were interpreted according to the Infevers (Internet Fevers) database (https://infevers.umai-montpellier.fr/web/), a curated international repository for autoinflammatory disease-associated variants.

For genotype–phenotype analyses, patients were additionally categorized according to exon involvement and allelic burden. Variants involving exon 10 were analysed separately. Patients were classified as monoallelic heterozygous, homozygous or compound heterozygous for exon-based analyses.

M694V, M680I, V726A and M694I were considered pathogenic exon 10-associated variants based on established literature and prior genotype–phenotype studies. In contrast, R202Q and E148Q were classified as low-penetrance variants with debated pathogenic significance.

### Statistical analysis

Statistical analyses were performed using IBM SPSS Statistics version 26.0 (IBM Corp., Armonk, NY, USA). Continuous variables were assessed for normality using the Shapiro–Wilk test and summarized as mean and standard deviation (s.d.) or median and interquartile range (IQR), as appropriate. Categorical variables were expressed as frequencies and percentages.

Comparisons between genotype groups were performed using Pearson’s χ² test or Fisher’s exact test for categorical variables and the Mann–Whitney *U*-test or Kruskal–Wallis test for continuous variables, depending on data distribution.

To reduce the risk of type I error arising from multiple subgroup comparisons, Bonferroni correction was applied where appropriate. Corrected significance thresholds were interpreted alongside nominal *P*-values in exploratory subgroup analyses.

Multivariable logistic regression analyses were conducted to identify independent predictors of early-onset disease, defined as symptom onset at ≤3 years of age. Hierarchical regression models were constructed to separately evaluate (i) the contribution of the most prevalent exon 10-associated variants (M694V, M680I and V726A) and (ii) the effect of exon 10 allelic burden (monoallelic *vs* biallelic involvement). Both models were adjusted for sex, family history of FMF and geographic region. Model calibration was assessed using the Hosmer–Lemeshow goodness-of-fit test, and model explanatory performance was evaluated using Nagelkerke’s *R*². Results were reported as odds ratios (ORs) with 95% CIs.

Given the controversial pathogenic significance of low-penetrance variants, primary genotype–phenotype analyses excluded isolated R202Q heterozygosity, while the robustness of findings related to isolated E148Q heterozygosity was evaluated through sensitivity analyses.

Graphical visualizations were generated using GraphPad Prism version 10.0 (GraphPad Software, Boston, MA, USA). Geographic distribution maps were created using ArcGIS Enterprise version 11.0 (Esri, Redlands, CA, USA).

### Ethical committee approval

The study protocol received approval from the Medical Research Ethics Committee of the ‘New Clinic’ Public Legal Entity (date/no: 15.05.2025: 013/25). Institutional permissions were obtained from all participating centres prior to data collection. Written informed consent was obtained from parents or legal guardians of all participants. The research was conducted in accordance with the principles of the Declaration of Helsinki and complied with all applicable local laws and regulations.

## Results

### Demographic and baseline characteristics

A total of 349 patients [211 (60.5%) boys; 138 (39.5%) girls] were included in the study. The median age at evaluation was 12 years (min–max: 2–18), with a median age at symptom onset of 4 years (min–max: 0.5–17) and a median age at diagnosis of 7 years (min–max: 1–17), resulting in a median diagnostic delay of 34 months (IQR: 12–60). The median follow-up period was 13.5 months (IQR: 12–33).

Parental consanguinity was reported in 78 (22.3%) patients, and 50 (14.3%) patients had a family history of FMF. Among comorbid conditions, allergies were present in seven (2.0%) patients, juvenile idiopathic arthritis (JIA) in 10 (2.9%) and immunoglobulin A vasculitis (IgAV) in nine (2.6%). Additionally, two patients had a history of appendectomy.

### Spectrum of *MEFV* variants

Exon 10 alterations emerged as the primary allelic contributors, accounting for the largest proportion of the *MEFV* variant spectrum identified in Azerbaijan. The most common *MEFV* variant patterns were M694V (*n* = 165, 47.3%), V726A (*n* = 70, 20.1%), R202Q (*n* = 69, 19.8%), E148Q (*n* = 62,17.8%) and M680I (*n* = 42, 12%) (Table 1).

**Table 1:** Spectrum and frequency of most common *MEFV* gene variants in the paediatric familial Mediterranean fever cohort.

**Variant**	*n* (%)
M694V/M694V	40 (11.5)
M694V/–	37 (10.6)
E148Q/–	30 (8.6)
V726A/–	25 (7.2)
R202Q/–	20 (5.7)
M694V/R202Q	18 (5.2)
M694V/V726A	15 (4.3)
M680I/M680I	15 (4.3)
M694V/E148Q	14 (4)
M680I/–	12 (3.4)
M694V/R761H	8 (2.3)
M680I/V726A	8 (2.3)
P369S/R408Q	8 (2.3)
M694V/M680I	6 (1.7)
A744S/–	6 (1.7)
M694I/M694I	4 (1.1)
M694I/V726A	4 (1.1)
V726A/V726A	4 (1.1)
R761H/–	4 (1.1)
M680I/R202Q	3 (0.9)
A744S/A744S	3 (0.9)
M680I/R761H	2 (0.6)
E148Q/E148Q	2 (0.6)
A744S/P369S	1 (0.3)
Complex variants	
M694V/V726A/R202Q	8 (2.3)
M694V/M694V+R202Q/R202Q	5 (1.4)
M694V/M694V+R202Q/−	4 (1.1)
P369S/R408Q/E148Q	4 (1.1)
M694V/M680I/R202Q	3 (0.9)
R202Q/R202Q+M694V/–	2 (0.6)

Regarding zygosity, 18.3% of patients were homozygous, 40% were heterozygous and 41.5% were compound heterozygous. Among exon 10-associated variants specifically, 18.9% were homozygous and 24.4% were heterozygous.

### Regional and genetic variant distribution in Azerbaijan

The geographic distribution of FMF cases was assessed by absolute counts and population-adjusted disease density. The largest numbers of patients were recorded in Lankaran (*n* = 53), Aran (*n* = 52) and Absheron (*n* = 45). Population-adjusted analysis revealed the highest disease density in West Zengezur (8.45 per 100 000), followed by Guba-Khachmaz (5.63 per 100 000) and Lankaran (5.61 per 100 000). Absheron showed the lowest disease density (1.39 per 100 000) (Fig. 1).

**Figure 1:**
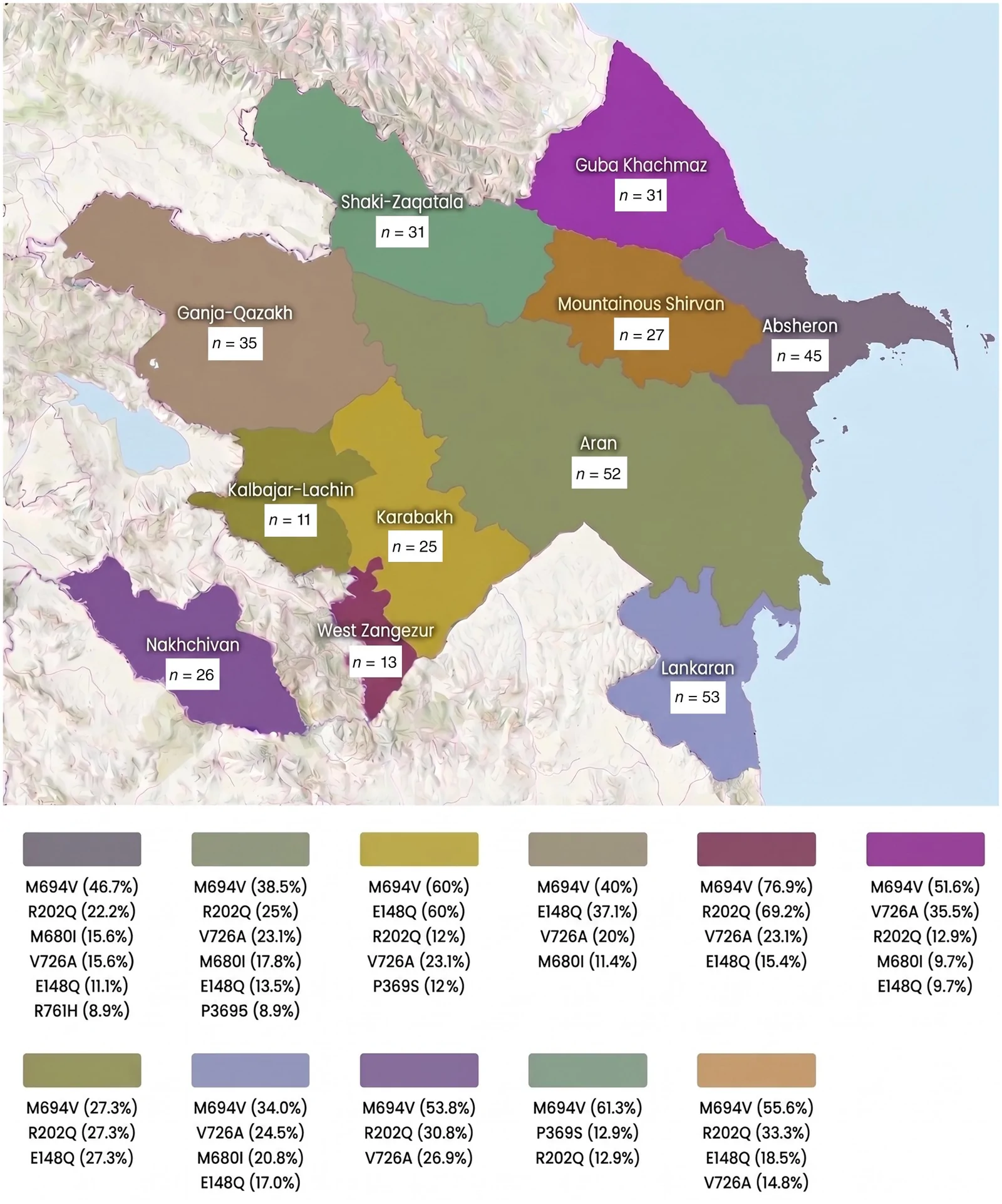
Regional distribution of *MEFV* variants among paediatric familial Mediterranean fever patients in Azerbaijan. Geographic distribution of *MEFV* gene variants is shown across the 11 major regions of Azerbaijan. The proportions of the most prevalent variants—including M694V, V726A, M680I, E148Q, R202Q and other regionally clustered variants—are shown for each region. Each bar represents the percentage of patients carrying a specific variant within that region, demonstrating substantial regional variability in *MEFV* mutation patterns

M694V remained the predominant *MEFV* variant throughout all 11 regions, although its frequency demonstrated marked geographic variation, ranging from 27.3% in Kalbajar–Lachin to 76.9% in West Zangezur (Fig. 1). Exon-based distribution patterns likewise revealed substantial regional heterogeneity. Monoallelic exon 10 variants were particularly prominent in Shaki-Zaqatala (35.7%), whereas homozygous exon 10 variants clustered more frequently in Absheron (24.3%) and Guba-Khachmaz (33.3%) (Fig. 2).

**Figure 2:**
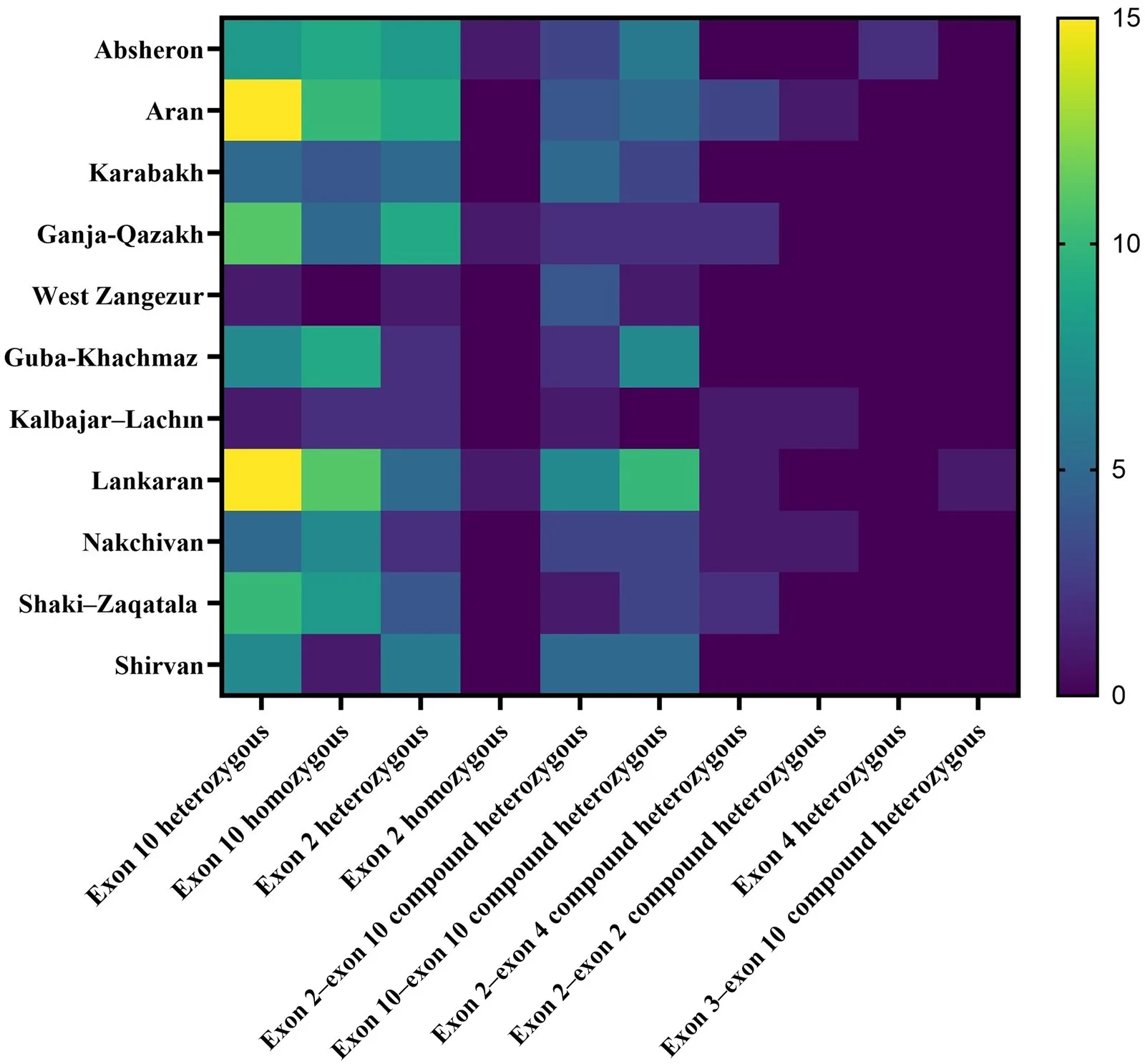
Regional heterogeneity and exon-based distribution of *MEFV* variants in Azerbaijan. Heatmap analysis representing the distribution of *MEFV* gene variants categorized by exon and zygosity across eleven administrative regions of Azerbaijan. The colour intensity scale (0–15) indicates the relative frequency of specific genotypic patterns within each region. The matrix highlights the prevalence of primary categories, such as exon 10 and exon 2 variants, alongside complex allelic combinations. Notably, exon 10 homozygous variants demonstrate marked clustering in Absheron and Guba–Khachmaz, while Lankaran and Aran exhibit high frequencies of exon 10 heterozygous patterns. Distinct regional signatures are further evidenced by the increased prevalence of exon 2 heterozygosity in Karabakh and Kalbajar–Lachın, and the concentration of diverse compound heterozygous combinations (e.g. exon 2–exon 10 and exon 10–exon 10) in regions like West Zangezur and Shirvan

### Clinical manifestations and genotype–phenotype relationships

After exclusion of patients with isolated R202Q heterozygosity (*n* = 20), the remaining cohort comprised 329 patients. Fever was the most frequent clinical manifestation, documented in 261 patients (79.3%), followed by abdominal pain, which occurred in 219 patients (66.6%). Musculoskeletal involvement was also common, with arthralgia reported in 64 patients (19.5%) and arthritis in 55 patients (16.7%). Less frequent manifestations included chest pain in 20 patients (6.1%) and erysipelas-like erythema in 10 patients (3%). Purpuric rash was observed in two patients (0.6%), both of whom had a concurrent diagnosis of IgAV (Table 2). Genotype–phenotype data are detailed in Supplementary Table S1.

**Table 2:** Comparison of clinical manifestations between exon 10 and non-exon 10 *MEFV* variant groups in paediatric familial Mediterranean fever.

Clinical findings	Exon-10 variants (*n* = 272)	Non-exon 10 variants (*n* = 57)	OR (95% CI)	*P*	Total
Age of symptom onset, median (min–max), years	4 (1–17)	5 (1–15)	—	**0.026** ^c^	4 (1–16)
Age of diagnosis, median (min–max), years	7 (1–17)	8 (1–16)	—	0.652^c^	7 (1–17)
Fever, *n* (%)	219 (80.5)	42 (73.7)	2.19 (1.25, 3.84)	0.207^a^	261 (79.3%)
Abdominal pain, *n* (%)	187 (69)	32 (56)	1.51 (0.90, 2.53)	0.08^a^	219 (67%)
Arthralgia, *n* (%)	51 (18.7)	13 (23)	0.81 (0.44, 1.51)	0.467^a^	64 (19.4%)
Arthritis, *n* (%)	44 (16.2)	11 (19.3)	0.80 (0.42, 1.53)	0.561^a^	55 (16.7%)
Vomiting, *n* (%)	20 (7.4)	4 (7)	0.94 (0.36, 2.42)	0.1^b^	24 (7.3%)
Chest pain, *n* (%)	18 (6.6)	2 (3.5)	1.29 (0.43, 3.91)	0.546^b^	20 (6%)
Diarrhea, *n* (%)	16 (5.9)	1 (1.8)	1.54 (0.44, 5.44)	0.325^b^	17 (5.2%)
Erysipelas-like erythema, *n* (%)	8 (2.9)	2 (3.5)	1.14 (0.24, 5.47)	0.686^b^	10 (3%)
IgA vasculitis, *n* (%)	2 (0.7)	0		1^b^	2 (0.6%)

a Comparisons between categorical variables were performed using Pearson’s χ² test when expected cell counts were adequate.

b Comparisons between categorical variables were performed using Fisher’s exact test when expected frequencies were <5.

c Continuous variables were compared using the Mann–Whitney *U*-test.

Bold values indicate statistical significance (*P* < 0.05). OR: odds ratio.

A comparative analysis was performed between patients harbouring at least one exon 10 variant of the *MEFV* gene (*n* = 272, 82.7%) and those without exon 10 involvement (*n* = 57, 17.3%). Fever was observed more frequently among patients with exon 10 variants compared with those without exon 10 involvement (80.5% *vs* 73.7%); however, this difference did not reach statistical significance (OR: 1.53; 95% CI: 0.74, 3.17; *P* = 0.207) (Table 2). No statistically significant differences were identified between the groups regarding other major FMF-related clinical manifestations.

When the comparison was limited exclusively to patients harbouring isolated exon 10 variants (homozygous, monoallelic heterozygous and compound heterozygous), a statistically significant variation in age at symptom onset (*P* *<* 0.001) and the age at diagnosis (*P* = 0.030) was observed between the monoallelic heterozygous and compound heterozygous groups. Similarly, age at symptom onset differed significantly among genotype categories, with compound heterozygous patients experiencing their first FMF-related symptoms at a markedly younger age compared with homozygous patients (*P = *0.007). With respect to clinical manifestations, abdominal pain was more frequently reported in both homozygous and compound heterozygous groups compared with monoallelic heterozygous individuals (*P = *0.040). No significant differences were observed among the groups regarding other symptoms.

To address the potential confounding effect of low-penetrance variants, we also performed a sensitivity analysis excluding patients harbouring monoallelic R202Q (*n* = 20) and E148Q (*n* = 30) variants. After exclusion of these patients, the principal genotype–phenotype associations remained materially unchanged, including the relationship between exon 10 variants and febrile attacks as well as the earlier disease onset observed in compound heterozygous patients.

A hierarchical logistic regression analysis was performed to identify factors associated with early-onset disease. After adjustment for sex, family history and region (block 1), the three most prevalent exon 10-associated variants (M694V, M680I and V726A) were entered into the model (block 2). Addition of the variant block significantly improved overall model fit (Δχ² = 12.47, df = 3, *P* = 0.006). Although the overall explanatory power of the model was modest (Nagelkerke *R*² = 0.060), model calibration was acceptable (Hosmer–Lemeshow *P* = 0.349).

Among the analysed variants, the presence of at least one M694V allele was independently associated with early-onset disease (OR = 2.10; 95% CI: 1.29, 3.43; *P* = 0.003). In contrast, M680I and V726A variants were not significantly associated with early disease onset.

To further evaluate the impact of exon 10 allelic burden on early disease onset, patients were categorized according to exon 10 zygosity status. After adjustment for sex, family history, region and age, the addition of exon 10 zygosity significantly improved model fit (Δχ² = 19.66, df = 2, *P* < 0.001). The overall model demonstrated good explanatory performance (Nagelkerke *R*² = 0.330) with acceptable calibration (Hosmer–Lemeshow *P* = 0.782).

Using biallelic exon 10 involvement as the reference category, both patients without exon 10 variants (OR = 0.22; 95% CI: 0.11, 0.45; *P* < 0.001) and those with monoallelic exon 10 variants (OR = 0.37; 95% CI: 0.20, 0.69; *P* = 0.002) showed significantly lower odds of early-onset disease. These findings indicate that biallelic exon 10 involvement represents the strongest genetic predictor of early disease onset in this cohort (Table 3).

**Table 3:** Hierarchical multivariable logistic regression analysis of factors associated with early-onset disease (≤3 years).

Variable	Adjusted OR (95% CI)	*P*-value
Model A: impact of specific variants^a^		
M694V variant (at least one allele)	**2.10 (1.29, 3.43)**	**0.003**
M680I variant	NS	>0.05
V726A variant	NS	>0.05
Model B: impact of genetic burden (zygosity)^b^		
Biallelic exon 10 involvement (reference)		
Monoallelic exon 10 variant	**0.37 (0.20, 0.69)**	**0.002**
No exon 10 variants	**0.22 (0.11, 0.45)**	**<0.001**

a Model A adjusted for sex, family history and region.

b Model B evaluates the effect of allelic burden (zygosity) compared with patients with no exon 10 variants.

Bold values indicate statistical significance (*P* < 0.05). NS: not significant; OR: odds ratio.

### Treatment response and adverse events

All patients received colchicine as the first-line therapy, with 97.4% achieving adequate disease control. Colchicine resistance was observed in 2.6% of patients (*n* = 9), of whom two (0.6% of the total cohort) required treatment escalation to anakinra. The remaining seven patients, who showed inadequate response despite receiving the maximum weight-based colchicine dose, were switched to an alternative internationally sourced colchicine preparation (compressed 1 mg tablets). IL-1 antagonist therapy was planned for these patients, and the necessary administrative procedures for treatment initiation were initiated. The colchicine-resistant subgroup was characterized by diverse *MEFV* allelic configurations, predominantly involving exon 10-associated variants and complex multiallelic combinations: E148Q heterozygous (*n* = 1), R202Q heterozygous/M694V homozygous (*n* = 2), R202Q/M694V/V726A compound heterozygous (*n* = 2), R202Q/M680I/M694V compound heterozygous (*n* = 2), M694V/V726A compound heterozygous (*n* = 1) and M694V heterozygous (*n* = 1).

Treatment-related adverse events occurred in 7.2% of the cohort (*n* = 25), occasionally with overlapping clinical manifestations in the same patient. Gastrointestinal adverse events predominated, particularly diarrhoea (*n* = 20) and nausea (*n* = 17). Transient elevations in liver transaminases were observed in 15 patients and resolved completely either spontaneously or after dose modification. No amyloidosis or serious adverse events occurred.

## Discussion

To our knowledge, this study constitutes the first large-scale, systematic investigation of the clinical phenotype, *MEFV* variant spectrum and regional distribution of FMF among paediatric patients in Azerbaijan, thereby addressing a substantial gap in the epidemiological understanding of FMF across the broader Caucasian genetic landscape.

Consistent with reports from Türkiye, Armenia, Georgia and Iranian Azerbaijani populations, M694V emerged as the predominant *MEFV* variant in our cohort (47.3%), reaffirming its central role in the genetic landscape of FMF across the broader Anatolian–Caucasian region [19–28]. This finding aligns with earlier data from Azerbaijan, in which a smaller predominantly adult FMF cohort demonstrated a similarly high frequency of the M694V allele (42.85%) [13]. This regional genetic pattern is further reinforced by findings from a large-scale analysis of 1330 individuals of Azerbaijani ethnicity from northwestern Iran, conducted over a 7-year period [28]. In this cohort, M694V was the most prevalent variant (42%), followed by E148Q (21%), V726A (19%), M680I (14%) and M694I (2%). The genotype distribution revealed that 18.27% of patients were homozygous, 27.82% compound heterozygous and 53.91% heterozygous for the studied variants [28]. Similarly, limited data from neighbouring Georgia identified M694V as the predominant variant both in a paediatric FMF cohort of 37 patients [26] and in a newborn screening study reporting an overall *MEFV* variant prevalence of 15.3% [27]. Collectively, these regional data demonstrate a genetic variant profile that closely parallels that of Turkish cohorts and our cohort, further underscoring the shared ancestral and genetic continuity across the Anatolian–Caucasian region.

In our cohort, elevated frequencies of V726A and R202Q contribute to a distinctive regional signature that partially overlaps with Mediterranean and Middle Eastern patterns but diverges from North African, Arab and Jewish subpopulations, indicating region-specific founder effects. Similar patterns exist in Türkiye (M694V, E148Q, M680I, V726A predominate) [24] and Israel, where M694V is most frequent in non-Ashkenazi Jews, E148Q in Ashkenazi Jews and V726A in Arabs [29, 30]. These findings suggest Azerbaijan shares the broader FMF genetic framework of neighbouring regions while exhibiting unique pattern of genetic variation shaped by its complex historical and ethnolinguistic background.

In addition to the predominance of M694V, our cohort demonstrated substantial regional heterogeneity in *MEFV* variant distribution across the eleven geographic regions of Azerbaijan. Although the overall distribution pattern supports the close genetic relationship between Azerbaijani populations and neighbouring Mediterranean and Near Eastern communities, marked regional variation was observed within the country itself. The frequency of the M694V allele ranged from 27.3% in Kalbajar–Lachin to 76.9% in West Zangezur, suggesting possible regional genetic structuring. Notably, the particularly high frequency observed in West Zangezur, a region geographically adjacent to Armenia, may reflect shared regional genetic backgrounds; however, such interpretations remain hypothesis-generating in the absence of dedicated population-genetic or haplotypic studies. Similarly, regional differences in disease density should be interpreted with caution, as they may reflect referral and diagnostic factors rather than genetic prevalence alone. Collectively, these findings further emphasize the complex and regionally diverse genetic architecture of FMF within the Caucasian population [31].

An important clinical finding of our study was the substantial diagnostic delay observed in Azerbaijani children with FMF. The median diagnostic delay of 34 months observed in our cohort represents a clinically relevant observation that warrants further attention. This prolonged delay likely reflects limited provider awareness of FMF, insufficient paediatric rheumatology infrastructure and a critically low number of paediatric rheumatologists in Azerbaijan, underscoring the urgent need for improved diagnostic pathways and expanded subspecialty access. Comparison with neighbouring cohorts underscores the broader regional significance of this finding. Tanatar *et al.* reported a mean diagnostic delay of 2.6 (2.8) years in a large Turkish paediatric cohort, while Kostik *et al.* documented a delay of 5.5 years among Crimean Turks [17, 18]. Öztürk *et al.* reported a median delay of 15 months in the one of the largest Turkish FMF series [24], and a study of Armenian children with FMF-associated vasculitis reflected a diagnostic delay of nearly 7 years [32]. The 34-month delay in our cohort is consistent with these regional data, reinforcing the value of our study as a foundational dataset for developing region-specific early diagnostic strategies across the Caucasian FMF belt.

Our genotype–phenotype analyses further support the pathogenic contribution of exon 10-associated *MEFV* variants to disease expression and early disease onset.

When analyses were restricted to isolated exon 10-associated genotypes, compound heterozygous patients exhibited significantly earlier symptom onset and diagnosis compared with monoallelic patients, while both homozygous and compound heterozygous patients demonstrated more frequent abdominal pain attacks. Previous studies have similarly linked exon 10-associated variants, particularly M694V-containing genotypes, with earlier disease onset and more severe inflammatory phenotypes characterized by recurrent fever and serositis [24, 33].

Importantly, multivariable hierarchical regression analyses further clarified the relative contribution of individual variants and exon 10 allelic burden to early disease onset. Specifically, the presence of at least one M694V allele independently increased the likelihood of symptom onset at ≤3 years of age, whereas M680I and V726A did not retain independent significance after adjustment. In parallel, the zygosity-based model demonstrated that biallelic exon 10 involvement represented the strongest genetic predictor of early-onset disease. Collectively, these findings suggest that early disease expression in Azerbaijani paediatric FMF is driven predominantly by exon 10-related genetic burden, particularly M694V-associated biallelic configurations, rather than by isolated single-variant effects alone.

Interpretation of genotype–phenotype associations in FMF remains challenging because of the debated pathogenic significance of low-penetrance variants such as E148Q [34, 35]. Despite uncertain pathogenicity in isolation, E148Q may exert an aggravating effect when paired with high-penetrant variants. Studies suggest M694V/E148Q genotypes may cause treatment-resistant disease [24], while E148Q can mediate dominant transmission with M694I or increase amyloidosis risk with V726A [36]. In support of this, Aktaş *et al.* recently reported that E148Q heterozygous patients did not exhibit a clinically distinct course compared with other FMF genotypes, and secondary amyloidosis developed in 7% of these patients, challenging the notion that E148Q should be regarded as a simple benign polymorphism [37]. In the present study, exclusion of patients with isolated R202Q or monoallelic E148Q variants did not materially alter the principal genotype–phenotype associations, particularly the relationship between exon 10-associated allelic burden and earlier disease onset. These findings support the robustness of our results while also suggesting that low-penetrance variants alone are unlikely to fully account for the observed early-onset phenotype.

Principal strengths of this study include its large sample size, systematic regional analysis, and comprehensive genotype–phenotype assessment within a previously undercharacterized population. These features provide a robust foundation for understanding FMF genetic epidemiology in the Caucasian region, and this dataset is poised to serve as a foundational reference for future epidemiological and genetic studies across the broader Caucasian FMF belt.

However, several limitations warrant consideration. The retrospective design and relatively short median follow-up period constrain our capacity to evaluate long-term outcomes, particularly the development of AA amyloidosis. Additionally, given the retrospective, referral-based nature of the cohort, referral bias cannot be entirely excluded as a contributor to the observed regional differences in disease density. The inclusion of variants of uncertain significance, such as R202Q and E148Q in monoallelic states, may have influenced the estimated prevalence of identified variants within our cohort. Nevertheless, these variants were deliberately included to provide comprehensive baseline genetic data, as the majority of affected patients exhibited clinically significant, treatment-responsive disease, offering insights into the phenotypic expression associated with these genotypes. Future prospective studies with extended follow-up are needed to assess long-term outcomes, validate low-penetrance variants functionally and determine precise disease prevalence.

This study provides the most comprehensive characterization of paediatric FMF in Azerbaijan to date, revealing a unique *MEFV* variant profile, with substantial regional heterogeneity and important genotype–phenotype correlations. Compound heterozygosity was associated with earlier symptom onset in univariate analyses, whereas the presence of at least one M694V allele remained the main independent predictor in multivariable models. Together, these findings may support a potential ‘second-hit’ effect of low-penetrance variants and highlight favourable therapeutic outcomes with early colchicine treatment. These results have important implications for genetic counselling, early diagnosis and personalized treatment strategies in this population and contribute to the broader understanding of FMF across genetically related populations in the Caucasus and Mediterranean regions.

## Supplementary Material

keag501_Supplementary_Data

## Data Availability

The data underlying this article will be shared on reasonable request to the corresponding author.
