# Supplementary material for: Familial Mediterranean fever in Azerbaijani children: a nationwide cohort study of genotype–phenotype association
Source: Rheumatology (Oxford). 2026 Sep 11;65(10):keag501. doi: 10.1093/rheumatology/keag501 (PMC13652145; doi:10.1093/rheumatology/keag501)
Supplement: Supplementary file 1 [file keag501_supplementary_data.docx]

**Supplementary Table S1***.* **Variant-Specific Clinical Associations in Pediatric FMF**

| **Variation** | **Abdominal pain** | **Fever** | **Arthritis** | **Arthralgia** | **ELE** | **Vomiting** | **Diarrhea** | **Chest pain** |
| --- | --- | --- | --- | --- | --- | --- | --- | --- |
| M694V/M694V  ( n=40) | 28 (70%) | 34 (85%) | 10 (25%) | 8 (20%) | 1 (2.5%) | 4 (10%) | 1(2.5%) | 1(2.5%) |
| M694V/-  ( n=37) | 20 (54 %) | 28 (76%) | 3 (8.1 %) | 5 (13.5%) | 1 (2.7%) | 2 (5.4%) | 1 (2.7%) | 1 (2.7%) |
| M680I / M680I  ( n=15) | 9 (60%) | 14 (93.3%) | 1 (6.7 %) | 2 (13.3%) | 11 (73 %) |  |  |  |
| M680I / -  ( n=12) | 6 (50%) | 10 (83.3%) | 2 (16.7%) | 2 (16.7%) |  | 1 (8.3%) |  | 1 (8.3%) |
| M694V / R761H  ( n=8) | 4 (50%) | 6 (75%) | 2 (25%) | 2 (25%) |  |  | 1 (12.5%) |  |
| M694V/ V726 A  ( n=15) | 10 (66.6%) | 12 (80%) |  |  |  | 1 (6.6%) |  | 1 (6.6%) |
| M694V/ R202Q  ( n=18) | 8 (44.4%) | 8 (44.4%) | 1 (5.6%) | 3 (17%) | 1 (5.6%) |  | 1 (5.6%) | 3 (17%) |
| M680I /V726A  ( n=8) | 7 (87.5%) | 8 (100%) | 1 (12.5) | 1 (12.5) |  | 2 (25%) | 2 (25%) | 1(12.5) |
| M694V / E148Q  ( n=14) | 2 (14.3%) | 4 (28.6%) |  |  |  |  | 1 (7.2%) | 2 (14.3%) |
| P369S/ R408Q  ( n=8) | 3 (37.5%) | 7 (87.5) | 2 (25%) | 2 (25%) |  | 1 (12.5%) |  |  |
| V726A / -  ( n=25) | 13 (52%) | 16 (64%) | 5 (20%) | 7 (28%) | 1 (4%) |  | 1 (4%) |  |
| E148Q / -  ( n=30) | 16 (53.3%) | 21 (70%) | 6 (20%) | 9 (30%) | 1 (3.3%) | 2 (6.7%) | 1 (3.3%) | 2(6.7%) |
